# Preoperative strain ultrasound elastography can predict occult central cervical lymph node metastasis in papillary thyroid cancer: a single-center retrospective study

**DOI:** 10.3389/fonc.2023.1141855

**Published:** 2023-04-12

**Authors:** Long Liu, Gang Li, Chao Jia, Lianfang Du, Qiusheng Shi, Rong Wu

**Affiliations:** ^1^ Department of Ultrasound, Shanghai General Hospital of Nanjing Medical University, Shanghai, China; ^2^ Department of Ultrasound, Shanghai General Hospital, Shanghai Jiao Tong University School of Medicine, Shanghai, China

**Keywords:** ultrasound, ultrasound elastography, prediction, lymph node metastasis, papillary thyroid carcinoma

## Abstract

**Objective:**

To determine whether preoperative ultrasound elastography can predict occult central cervical lymph node metastasis (CCLNM) in patients with papillary thyroid cancer.

**Methods:**

This retrospective study included 541 papillary thyroid cancer patients with clinically negative lymph nodes prior to surgery between July 2019 and December 2021. Based on whether CCLNM was present on postoperative pathology, patients were categorized as CCLNM (+) or CCLNM (-). Preoperative clinical data, conventional ultrasound features, and ultrasound elastography indices were compared between the groups. Univariate and multivariate logistic regression analysis were performed to identify the independent predictors of occult CCLNM.

**Results:**

A total of 36.60% (198/541) patients had confirmed CCLNM, while 63.40% (343/541) did not. Tumor location, bilaterality, multifocality, echogenicity, margin, shape, vascularity, capsule contact, extrathyroidal extension, aspect ratio, and shear wave elasticity parameters were comparable between the groups (all *P* > 0.05). Univariate analysis showed statistically significant differences between the two groups in age, sex, tumor size, calcification, capsule invasion, and strain rates ratio in strain ultrasound elastography (all *P* < 0.05). In multivariate logistic regression analysis, the independent predictors of occult CCLNM were age (OR = 0.975, 95% CI = 0.959-0.991, *P* = 0.002), sex (OR = 1.886, 95% CI = 1.220-2.915, *P* = 0.004), tumor size (OR = 1.054, 95% CI = 1.014-1.097, *P* = 0.008), and strain rates ratio (OR = 1.178, 95% CI = 1.065-1.304, *P* = 0.002).

**Conclusion:**

Preoperative strain ultrasound elastography can predict presence of occult CCLNM in papillary thyroid cancer patients and help clinicians select the appropriate treatment strategy.

## 1 Introduction

The incidence of thyroid cancer has increased significantly in many countries, with papillary thyroid carcinoma (PTC) being the most common pathological type reported (1, 2). Central cervical lymph node metastasis (CCLNM) is detected by postoperative pathological examination in 48% of patients with PTC (3), while about 50% of patients have occult CCLNM (4, 5). CCLNM is associated with risk of disease recurrence (6, 7); however, it does not affect survival and prognosis in clinically lymph node- negative (cN0) PTC patients (8, 9), and so prophylactic central lymph node dissection (CLND) is not mandated in the latest guidelines and research (10–13). For low-risk PTC patients, minimally invasive thermal ablation strategies or active surveillance are currently favored (14–17). Methods to preoperatively predict presence of occult CCLNM in cN0 patients can help clinicians make appropriate treatment decisions.

Previous studies have identified extrathyroidal extension (ETE), vascular invasion, and pathologic type as indicators of possible occult CCLNM (18–20), but the use of postoperative pathological indicators has limited value for guiding treatment decisions. Preoperative indicators of occult CCLNM would be far more useful.

Preoperative conventional ultrasonography can detect lateral cervical lymph node metastases in PTC patients (21), but its sensitivity for diagnosing CCLNM is low due to interference from gas and bones (22, 23). Ultrasound elastography (UE) can help distinguish between benign and malignant thyroid nodules (24) and also determine the aggressiveness of PTCs by identifying features such as cervical lymph node metastasis (25–27) and ETE (28). However, the use of UE to predict occult CCLNM in PTC patients has not been fully investigated. The aim of this retrospective study was to identify the clinical and preoperative conventional ultrasonography and UE features that could predict the presence of occult CCLNM in cN0 PTC patients.

## 2 Materials and methods

This retrospective study was conducted between July 2019 and December 2021 after obtaining approval from the hospital’s ethics committee. The need for informed consent was waived.

### 2.1 Patients

**Figure 1:**
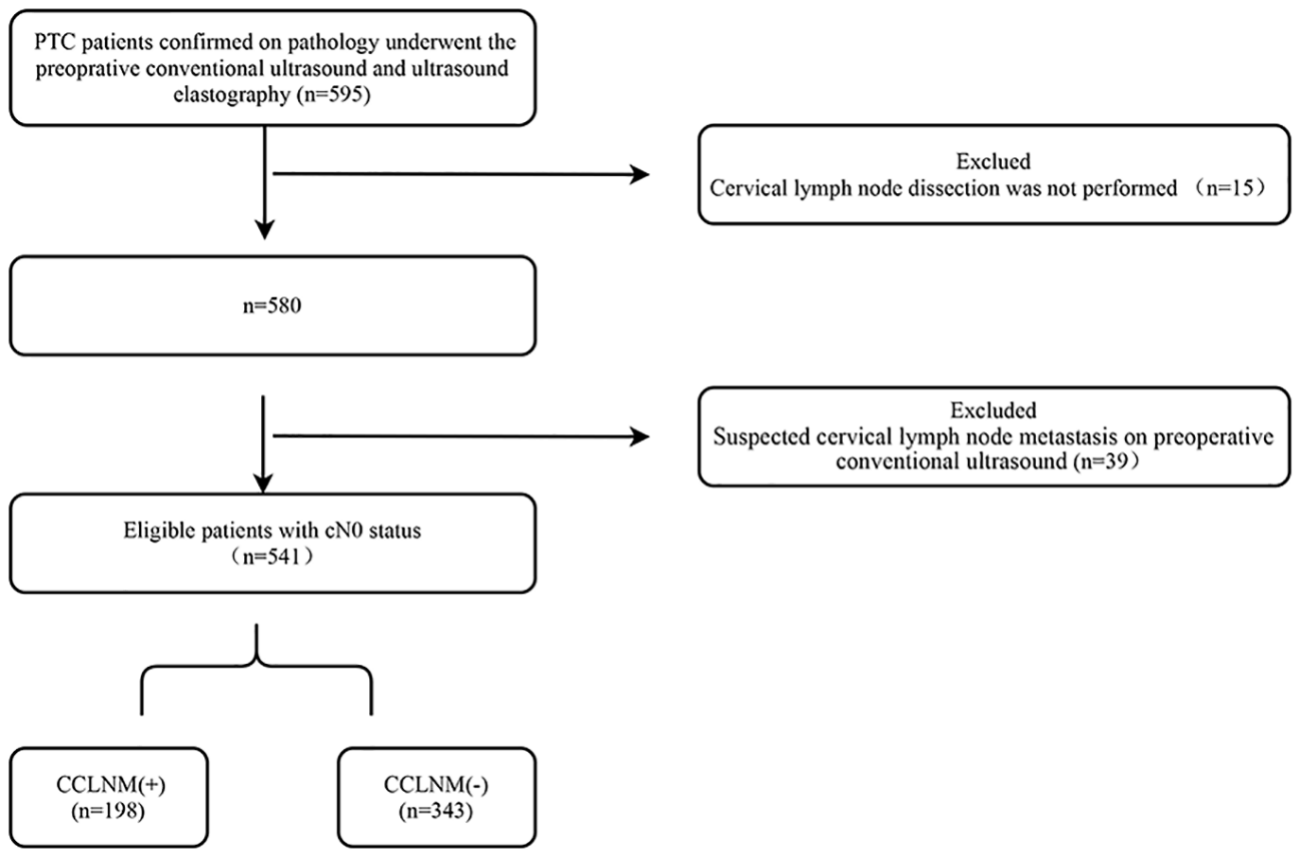
Flow chart showing patient selection process. PTC, papillary thyroid cancer; CCLNM, central cervical lymph nodes metastasis; cN0, clinically lymph node-negative.

The study sample was selected from among 595 patients who underwent surgery after fine needle aspiration cytology–confirmed diagnosis of PTC. The inclusion criteria were 1) preoperative conventional ultrasonography and UE examination performed before fine needle aspiration cytology and 2) diagnosis of PTC confirmed by postoperative pathological examination. Patients who did not undergo CLND and those with suspected cervical lymph node metastasis on preoperative conventional ultrasonography were excluded. On conventional ultrasound, the features suspicious of cervical lymph node metastasis include rounded shape, increased cortical echogenicity, cystic changes, microcalcification, loss of the fatty hilum, and peripheral vascularity. Among the 595 patients, 39 had suspected lymph node metastases on preoperative conventional ultrasound. These patients were excluded from the study regardless of whether cytology of the lymph nodes had been performed. Among the 39 patients, 26 with suspicious lymph nodes in area II, III, and IV; while 19 underwent fine needle aspiration for confirmation, 7 did not undergo fine needle aspiration because the lymph nodes were large and showed typical ultrasound features of metastasis. Meanwhile, another 13 patients with suspicious lymph nodes in area VI, did not undergo fine needle aspiration because central CLND is routinely performed in our hospital. Thyroglobulin measurement was not routinely performed in suspected lymph nodes detected by routine preoperative ultrasound at our clinical center. The flowchart in **Figure 1** shows the patient selection process. A total of 541 patients (with 594 PTCs) met the eligibility criteria and were included in this study. While 388 patients had a solitary PTC, 153 patients had multiple PTCs. For patients with multiple PTCs, the largest tumor was selected for evaluation. Among the 541 patients, 198 (36.60%) patients (60 males, 138 females; age range, 17-77 years, median age, 40 years) had postoperative pathology confirmed CCLNM and 343 (63.40%) patients (59 males, 284 females; age range, 22-75 years, median age, 45 years) did not have CCLNM; they were classified as the CCLNM (+) and CCLNM (-) groups, respectively. Tumor size ranged from 2.50 mm to 38.40 mm (median, 8.35 mm) in the CCLNM (+) group, and 2.00 mm to 44.10 mm (median, 6.60 mm) in the CCLNM (-) group.

### 2.2 Conventional ultrasonography

Ultrasound examinations were performed using an Aplio i900 ultrasound scanner (Cannon Medical Systems Corp, Tochigi-ken, Japan) equipped with a high-frequency linear transducer (frequency range, 5-18 MHz). During examination, patients lay supine with a pillow under the shoulder to keep the neck extended. Gain, depth, and time-gain compensation were optimized before the examination. The entire thyroid and bilateral cervical lymph nodes were first examined, and then the tumors were evaluated in both longitudinal and transverse views.

On gray-scale ultrasound, the following tumor characteristics were evaluated: echogenicity, margin, shape, calcification, capsule contact, capsule invasion, ETE, and aspect ratio. Tumor echogenicity was classified as hypoechoic, hyperechoic, or isoechoic as compared to the echogenicity of surrounding thyroid parenchyma (STP). The margin was classified as well-defined or ill-defined. Shape was classified as regular or irregular. Calcification in the tumor was classified as no calcification, microcalcification (bright punctate echoes measuring <1 mm), macrocalcification, and mixed type of calcification (combined coarse calcification and microcalcification). Capsule contact was defined as the tumor being in contact with the thyroid capsule. Capsule invasion was considered present when the thyroid capsule echogenicity was interrupted. ETE was defined as tumor extension beyond the thyroid capsule. Aspect ratio was defined as the ratio of anterior–posterior diameter to the left–right diameter of the tumor in cross-sectional view.

Color Doppler ultrasound was performed to assess tumoral and parenchymal vascularity; four patterns were defined: 1) type I, none, 2) type II, abundant peritumoral blood flow and slight intratumoral blood flow, 3) type III, slight peritumoral and intratumoral blood flow; and 4) type IV, abundant intratumoral blood flow.

### 2.3 UE examination

Two modes of UE were used: strain ultrasound elastography (SUE) and shear wave elasticity (SWE). SUE was performed first, followed by SWE, with the patient in the same position as for conventional ultrasound examination. SUE was performed in the long axis of the tumor. After the tumor was displayed on gray-scale ultrasound, the SUE mode was activated. The size of the sampling frame was adjusted to include as much as possible of both tumor and STP. The transducer was used to apply periodic external compression while SUE images of the tumor were acquired. A quality control frame was used to monitor the compression. The frequency and magnitude of the applied force, as well as the range scale of the elasticity, were adjusted according to the quality control frame. Five still SUE images with good quality control were stored. A continuum of colors (from red to green and blue) in SUE images indicates different levels of tissue stiffness (from soft to medium and hard). For SWE examination, the transducer was placed gently on the neck, and the “one-shot” method was chosen to acquire five SWE images for quantitative analysis. SWE provides two values: elasticity (kPa) and shear wave velocity (SWV, m/sec). All images were stored in the ultrasound instrument.

*SUE measurement*

**Figure 2:**
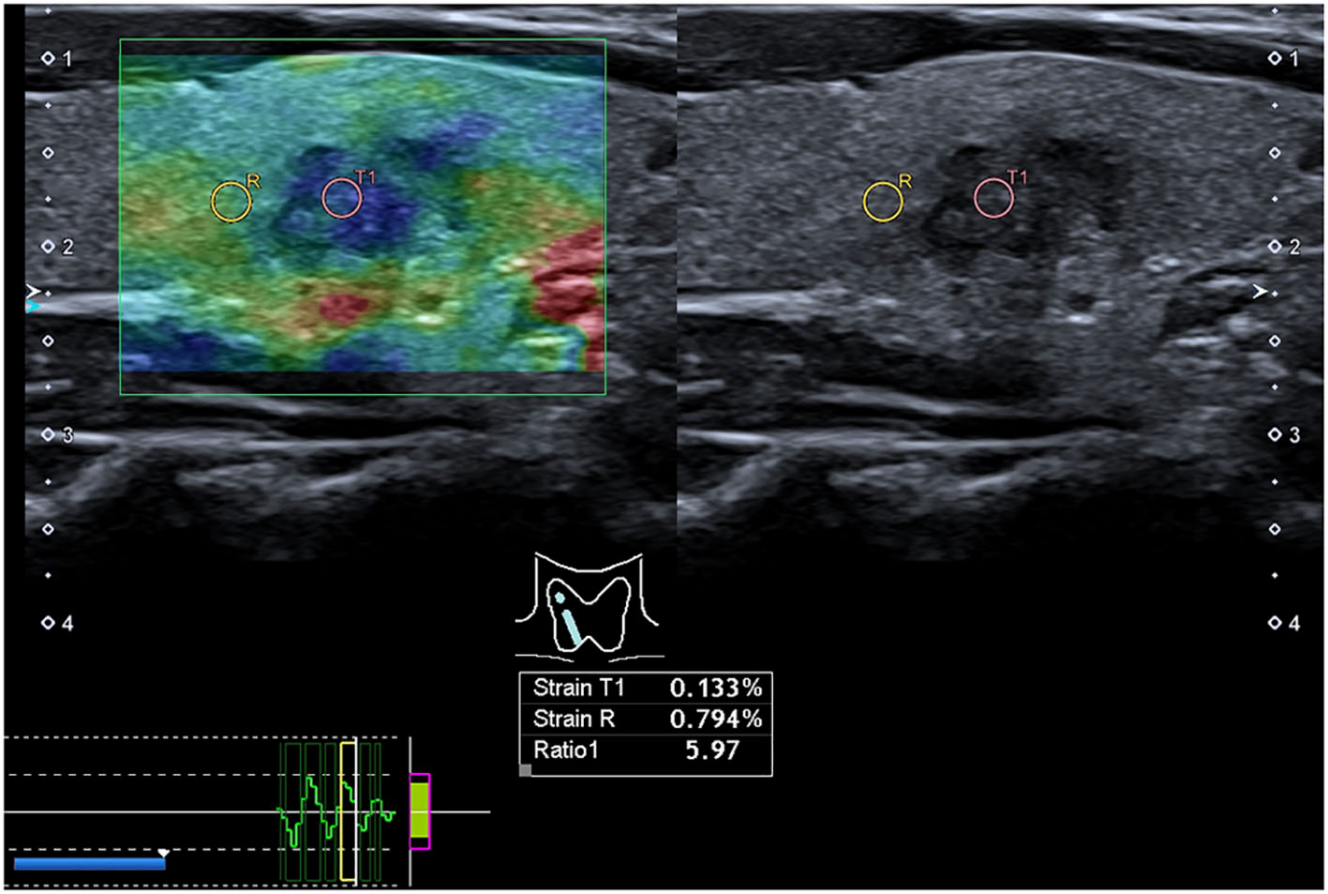
Preoperative strain ultrasound elastography images. Preoperative strain ultrasound elastography of a patient with papillary thyroid cancer in the right lobe of the thyroid. The image on the left shows the hardness distribution of tumor tissue (T1) and surrounding thyroid parenchyma (R) in a green sampling box. The color distribution is from red to blue (blue areas indicate hard areas, and red areas indicate soft areas). The right image shows a gray-scale ultrasound image corresponding to the strain ultrasound elastography image.

First, a circular region of interest (ROI) with diameter of 2 mm was placed inside the tumor as the target region. For tumors with marked uneven elasticity, the ROI was placed in the hardest region within the tumor. Then, a similar ROI was placed in the STP at the same depth as the ROI in the tumor. The system automatically calculated the strain rate value of the tumor and the STP. Strain rate ratio (SRR) of STP to the tumor was calculated (**Figure 2**). A higher SRR value indicates a stiffer tumor. Five SRR values were obtained from five previously stored SUE images, and the median value was used for analysis.

*SWE measurement*

**Figure 3:**
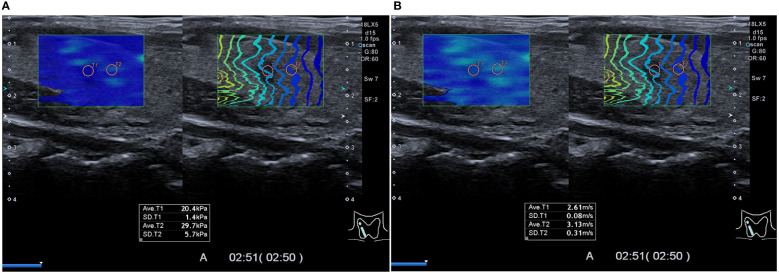
Preoperative shear wave elasticity images. Preoperative shear wave elasticity image of a patient with papillary thyroid cancer in the right lobe of the thyroid. Results of two types of shear wave elasticity measurement are shown in **(A, B)** as kPa and m/s, respectively. The region of interest was placed on the tumor (T1) and the surrounding thyroid parenchyma (T2), respectively, in the green sampling box. On the left, the color indicates the hardness of the tissue, and on the right, the contour lines depict shear wave arrival times at different points.

For SWE, the circular ROI was of the same size and depth as for SUE. Ten SWE parameters were recorded (**Figure 3**): tumor elasticity value (SWE_tumor), standard deviation of tumor elasticity value (SWESD_tumor), STP elasticity (SWE_STP), standard deviation of STP elasticity (SWESD_STP), elasticity ratio of tumor to the STP (SWE_ratio); tumor SWV (SWV_tumor), standard deviation of SWV (SWVSD_tumor), STP SWV (SWV_STP), standard deviation of STP SWV (SWVSD_STP), and SWV ratio of tumor to the STP (SWV_ratio). The median of each parameter was used for the final analysis.

### 2.4 Clinical data

Data on patient age, sex, tumor size, tumor location (upper, middle, lower, whole [occupying at least two parts of thyroid], and isthmus), bilaterality, multifocality, surgical procedure, and pathological findings were obtained from the hospital’s information management system.

### 2.5 Reliability of conventional ultrasonography indices

Qualitative information from conventional ultrasonography was assessed independently by two sonographers (each with more than 10 years of experience in thyroid ultrasonography) who were blinded to the pathological results. Inter-reader agreement was used to evaluate the reliability of the conventional ultrasonography indices.

### 2.6 Statistical analysis

Statistical analysis was performed using SPSS 23.0 (IBM Corp., Armonk, NY, USA). Qualitative data were compared between groups using the chi-square test or Fisher exact probability method, while quantitative data were compared using the *t*-test or a nonparametric rank sum test, according to the normality of data distribution. One-way analysis of variance was first conducted. Variables found to be significantly (at *P* < 0.05) associated with occult CCLNM in univariate analysis were entered into multivariate binary logistic regression to identify the independent predictors of occult CCLNM. Inter-reader agreement was assessed using Cohen’s *κ* statistics. For all tests, *P* < 0.05 was considered to indicate statistically significant difference.

## 3 Results

### 3.1 Surgery and pathological findings

Among the 541 patients, 159 (29.39%) underwent total thyroidectomy, 358 (66.17%) underwent unilateral thyroid lobectomy and isthmusectomy, 4 (0.74%) underwent bilateral partial thyroidectomy and isthmusectomy, and 20 (3.70%) underwent unilateral thyroidectomy and contralateral partial thyroidectomy. In addition, 182 (33.64%) patients underwent bilateral central CLND; while 180 of these patients underwent bilateral central CLND only, 2 patients underwent unilateral lateral CLND in addition to bilateral central CLND. A total of 359 (66.36%) patients underwent unilateral central CLND.

### 3.2 Baseline clinical features

**Table 1** summarizes the baseline data. Compared to CCLNM (-) patients, CCLNM (+) patients were significantly younger (*P* < 0.001), more likely to be male (*P* < 0.001), and to have larger tumors (*P* < 0.001). Tumor location, bilaterality, and multifocality were not different between the two groups (*P* =0.398, 0.950, 0.691, respectively).

**Table 1:** Baseline data of patients with clinically lymph node-negative papillary thyroid cancer (n = 541).

Parameter	CCLNM (-) n = 343	CCLNM (+) n = 198	Statistic value	*P*
Age, y, median (IQR)	45 (18)	40 (17)	26791^b^	<0.001
Sex, n (%)				
Male	59 (17.20)	60 (30.30)	12.559^a^	<0.001
Female	284 (82.80)	138 (70.70)		
Size, mm, median (IQR)	6.60 (4.30)	8.35 (6.23)	24616^b^	<0.001
Location, n (%)				
Upper	43 (12.54)	20 (10.10)	4.058^a^	0.398
Middle	176 (51.31)	96 (48.48)		
Lower	83 (24.19)	51 (25.76)		
Whole	10 (2.92)	12 (6.06)		
Isthmus	31 (9.04)	19 (9.60)		
Bilaterality, n (%)				
Unilateral	290 (84.55)	167 (84.34)	0.004^a^	0.950
Bilateral	53 (15.45)	31(15.66)		
Multifocality, n (%)				
No	248 (72.30)	140 (70.71)	0.158^a^	0.691
Yes	95 (27.70)	58 (29.29)		

CCLNM, central cervical lymph node metastasis; (-), absence of central cervical lymph nodes metastasis; (+), presence of central cervical lymph nodes metastasis; IQR, interquartile range.

^a^Chi‐square test.

^b^Mann‐Whitney U test.

### 3.3 Conventional ultrasonography and UE features associated with CCLNM

**Figure 4:**
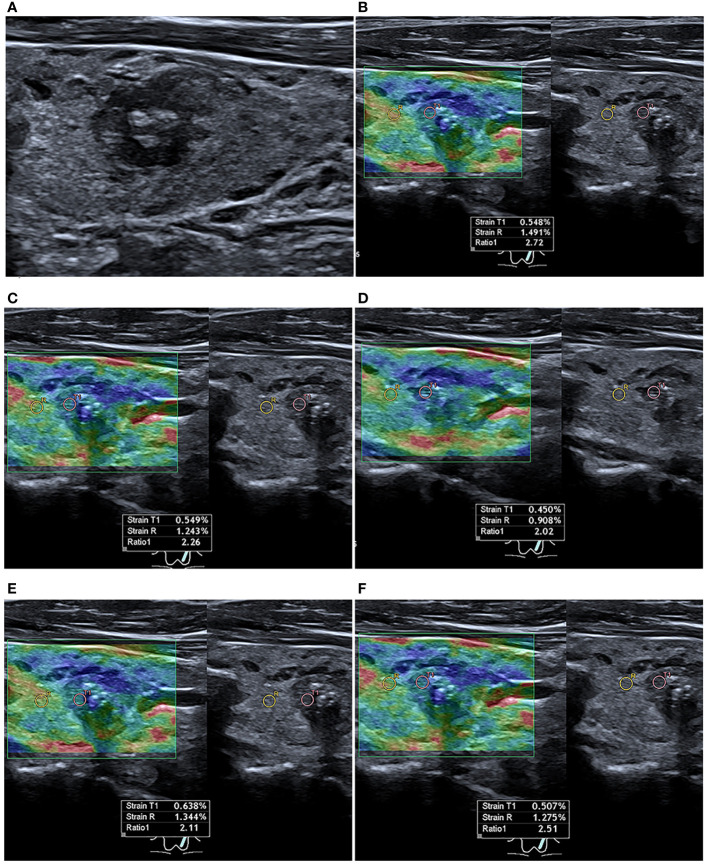
Conventional and strain ultrasound elastography of a 51-year-old male PTC patient with occult central cervical lymph node metastasis. **(A)** Conventional ultrasound shows a hypoechoic nodule in the lower left lobe of the thyroid gland with a maximum diameter of 12.30 mm. **(B–F)** Five strain ultrasound elastography images were obtained, with strain ratios of 2.72, 2.26, 2.02, 2.11, and 2.51, respectively.

**Figure 5:**
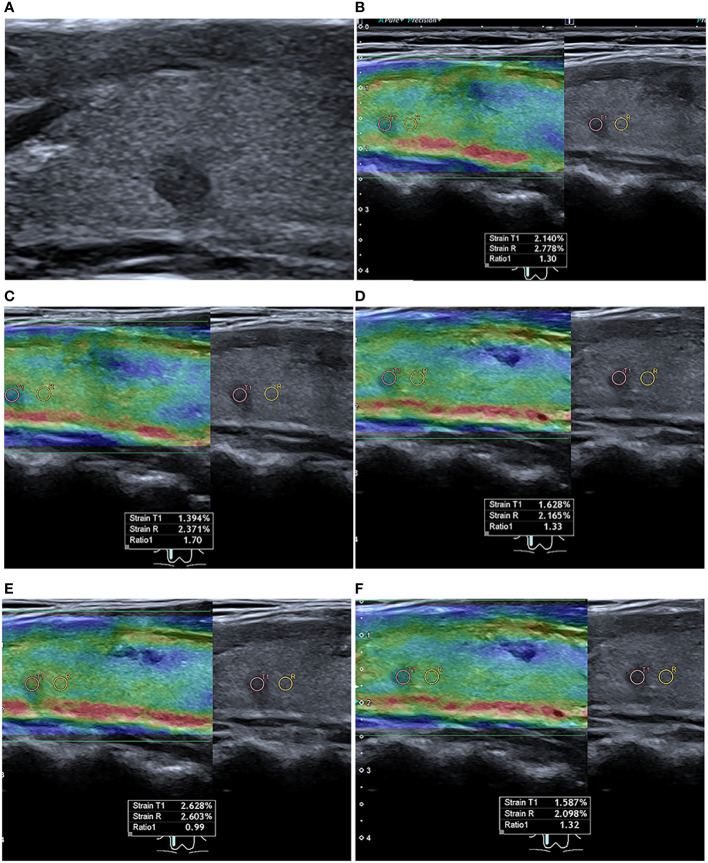
Conventional and strain ultrasound elastography of a 45-year-old female PTC patient without occult central lymph node metastasis. **(A)** Conventional ultrasound shows a hypoechoic nodule in the upper right lobe of the thyroid gland with a maximum diameter of 4.70 mm. **(B–F)** Five strain ultrasound elastography images were obtained, with strain ratios of 1.30, 1.70, 1.33, 0.99, and 1.32, respectively.

On conventional ultrasonography, calcification pattern was significantly different between the CCLNM (+) and CCLNM (-) groups (*P* = 0.006). Capsule invasion was more common in CCLNM (+) patients than in CCLNM (-) patients (*P* = 0.043). Tumor echogenicity, margin, shape, vascularity, capsule contact, ETE, and aspect ratio were comparable between the two groups (*P* = 0.132, 0.096, 0.544, 0.065, 0.801, 0.328, 0.174, respectively). On UE, SRR was significantly higher in the CCLNM (+) group than the CCLNM (-) group (*P* < 0.001) (**Figures 4**, **5**). All SWE parameters were comparable between the two groups (all *P* > 0.05; **Table 2**).

**Table 2:** Univariate analysis of the association between ultrasound features and occult CCLNM.

Ultrasound features	CCLNM (-), n = 343	CCLNM (+), n = 198	Statistic value	*P*
Echogenicity, n (%)			4.129^c^	0.132
Hypoechoic	320 (93.29)	175 (88.38)		
Hyperechoic	3 (0.87)	4 (2.02)		
Isoechoic	20 (5.83)	19 (9.60)		
Margin, n (%)			2.769^a^	0.096
Well-defined	148 (43.15)	71 (35.86)		
Ill-defined	195 (56.85)	127 (64.14)		
Shape, n (%)			0.368^a^	0.544
Regular	139 (40.52)	75 (37.88)		
Irregular	204 (59.48)	123 (62.12)		
Calcification, n (%)			12.435^a^	0.006
No calcification	155 (45.19)	66 (33.33)		
Microcalcification	122 (35.57)	97 (48.99)		
Macrocalcification	39 (11.37)	15 (7.58)		
Mixed type	27 (7.87)	20 (10.10)		
Vascularity, n (%)			7.238^a^	0.065
Type I	110 (32.07)	43 (21.72)		
Type II	30 (8.75)	16 (8.08)		
Type III	183 (53.35)	125 (63.13)		
Type IV	20 (5.83)	14 (7.07)		
Capsule contact, n (%)			0.063^a^	0.801
Absent	97 (28.28)	54 (27.27)		
Present	246 (71.72)	144 (72.73)		
Capsule invasion, n (%)			4.109^a^	0.043
Absent	234 (68.22)	118 (59.60)		
Present	109 (31.78)	80 (40.40)		
Extrathyroidal extension, n (%)			0.957^a^	0.328
Absent	282 (82.22)	156 (78.79)		
Present	61 (17.78)	42 (21.21)		
Aspect ratio, median (IQR)	1.05 (0.36)	1.02 (0.32)	31574.5^b^	0.174
Strain rates ratio, median (IQR)	1.68 (0.88)	2.35 (1.26)	20753.00^b^	<0.001
SWE_tumor, kPa, median (IQR)	33.50 (25.05)	33.50 (18.80)	33989.50^b^	0.970
SWESD_tumor, kPa, median (IQR)	6.40 (5.18)	6.40 (4.93)	33773.50^b^	0.872
SWE_STP, kPa, median (IQR)	26.20 (14.75)	26.20 (13.18)	33304.50^b^	0.668
SWESD_STP, kPa, median (IQR)	4.30 (2.90)	4.30 (2.90)	33519.50^b^	0.760
SWE_ratio, median (IQR)	1.30 (0.58)	1.30 (0.57)	33070.00^b^	0.574
SWV_tumor, m/s, median (IQR)	3.33 (1.09)	3.33 (0.85)	33453.00^b^	0.731
SWVSD_tumor, m/s, median (IQR)	0.31 (0.18)	0.31 (0.20)	33756.50^b^	0.864
SWV_STP, m/s, median (IQR)	2.93 (0.68)	2.92 (0.75)	33193.00^b^	0.623
SWVSD_STP, m/s, median (IQR)	0.24 (0.13)	0.24 (0.10)	34046.00^b^	0.995
SWV_ratio, median (IQR)	1.14 (0.23)	1.14 (0.21)	33149.00^b^	0.605

CCLNM, central cervical lymph nodes metastasis; (-), absence of central cervical lymph nodes metastasis; (+), presence of central cervical lymph nodes metastasis; SWESD, standard deviation of shear wave elasticity; STP, surrounding thyroid parenchyma; SWV, shear wave velocity; SWVSD, standard deviation of shear wave velocity; SWE, shear wave elasticity; IQR, interquartile range.

^a^Chi‐square test.

^b^Mann‐Whitney U test.

^c^Fisher exact probability method.

### 3.4 Multivariate logistic regression analysis

**Figure 6:**
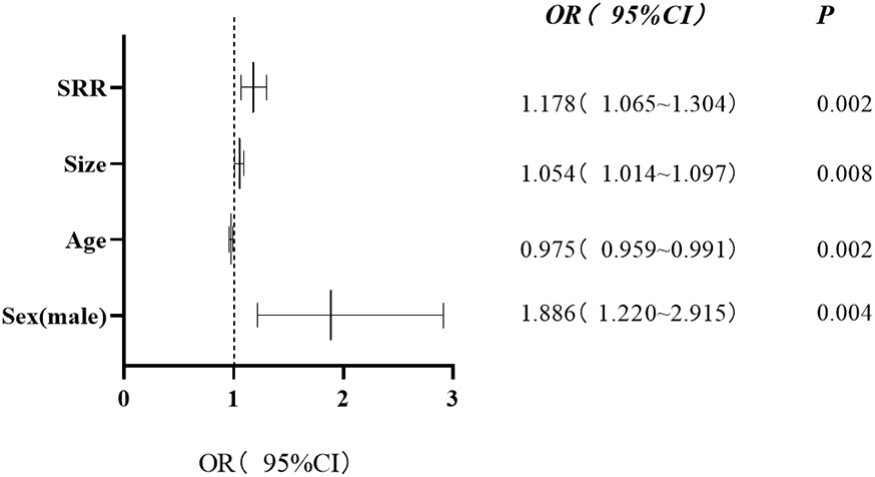
Forest plot showing the results of multivariate regression analysis. Four independent risk factors of occult central cervical lymph nodes metastasis were identified. The x-axis shows the relative odds ratio (OR) and 95% confidence interval (CI) of each covariate. OR (95% CI) and *P* values are presented on the right. SRR, strain rate ratio.

In binary logistic regression analysis, the independent predictors of occult CCLNM were age (OR = 0.975, 95% CI: 0.959-0.991, *P* = 0.002), sex (OR = 1.886, 95% CI: 1.220-2.915, *P* = 0.004), tumor size (OR = 1.054, 95% CI: 1.014-1.097, *P* = 0.008), and SRR (OR = 1.178, 95% CI = 1.065-1.304, *P* = 0.002) (**Figure 6**).

### 3.5 Receiver operating characteristic curve analysis

Based on the receiver operating characteristic curve analysis, the optimal cutoff values of continuous predictor variables were age 34 years, tumor size 9.95mm, and SRR 2.00. The sensitivity, specificity, and area under the curve of patient age, tumor size, and SRR for predicting occult CCLNM were 0.323, 0.837, 0.606; 0.409, 0.813, 0.638; and 0.656, 0.706, 0.694, respectively.

### 3.6 Inter-reader agreement

Inter-reader agreement was excellent for tumor echogenicity, margin, shape, calcification, capsule contact, capsule invasion, and ETE (*κ* range, 0.828-0.915), while it was good for vascularity (*κ* = 0.772).

## 4 Discussion

This study retrospectively evaluated the preoperative clinical information, conventional ultrasonography, and UE data of cN0 PTC patients to identify the predictors of occult CCLNM. The results showed that the age <34 years, male sex, SRR >2.00 and tumor size >9.95 mm were independent predictors of occult CCLNM.

Higher SRR indicates a harder PTC. In this study, SRR was significantly higher in the CCLNM (+) group than in the CCLNM (-) group, and multivariate analysis showed that it was an independent predictor of occult CCLNM. Our results are similar to those of Huang et al., who reported that elasticity score on SUE was independently associated with CCLNM (29). They used a semi-quantitative parameter, whereas we preferred to use the quantitative SRR. Quantitative parameters are more objective. It is possible that PTC hardness is related to aggressiveness. Previous studies have demonstrated correlation between the hardness of PTC and its aggressiveness, with higher hardness being related to greater probability of CCLNM and ETE (25, 28). Based on the results of this study, SRR >2.00 suggests likelihood of occult CCLNM.

We also examine the value of SWE for predicting occult CCLNM preoperatively, but found that none of the SWE parameters were independently related to risk for occult CCLNM. In contrast, a previous study found that SWE could be used to predict presence of occult CCLNM (30). The contradictory results may be because different SWE parameters were used in the two studies; they used the maximum SWE value (Emax), whereas we used the median value. Compared to SRR, SWE parameters are relatively susceptible to factors such as tumor size, tumor depth, pre-compression, neck morphology, fibrosis from previous neck surgery, and so on (31), which is probably why we could not demonstrate any predictive value for these parameters.

Age was an independent predictor of occult CCLNM in this study, with younger patients being more likely to have occult CCLNM. Our result is consistent with previous reports (32, 33). The cutoff age of 34 years in this study is close to the 36 years reported by Xu et al. (33).

This study found that male patients were significantly more likely to have occult CCLNM. The result was consistent with previous reports (32–34). Female patients have a greater tendency to develop PTC, which may be related to estrogen, but male patients appear to be more likely to develop occult CCLNM (35).

Another independent risk factor for occult CCLNM in this study was tumor size. Patients with larger tumors were more likely to have occult CCLNM (33, 34). The cutoff size identified in this study was 9.95 mm, which similar to the 8 mm reported by Xu et al. (33). According to our study, the risk of occult CCLNM was higher in patients with large non-micro-PTC than in those with micro-PTC. Hence, a conservative treatment strategy such as minimally invasive ablation therapy or active surveillance should be considered for micro-PTC patients.

This study has limitations. First, the retrospective single-center design makes it prone to selection biases. Second, most tumors in this study were small (median size, 8 mm), so the study results might not apply to patients with large tumors. Third, on SUE images, the PTCs were not uniformly hard, especially the larger PTCs, so the position of the ROI might have influenced the result. We chose the stiffest area on SUE images (the blue area) for measurement.

## 5 Conclusion

In patients with cN0, PTC, UE, especially SUE, might help clinicians identify presence of occult CCLNM preoperatively and allow selection of the most appropriate treatment strategy. Further multicenter studies with large sample sizes are needed to confirm the findings of this study.

## Data availability statement

The raw data supporting the conclusions of this article will be made available by the authors, without undue reservation.

## Ethics statement

The studies involving human participants were reviewed and approved by The ethics committee of Shanghai General Hospital. Written informed consent from the participants’ legal guardian/next of kin was not required to participate in this study in accordance with the national legislation and the institutional requirements.

## Author contributions

LL and RW conceived the idea and designed the research. LL, GL, CJ participated in the data collection and image analysis. LL analyzed the data. LL participated in manuscript preparation. LD and QS participated in methodology support. All authors contributed to the work and approved the submission.
